# Long-Term Clinical and Radiographic Osseointegration Outcomes of a Highly Porous 3D-Printed Titanium Collar Compared with a Hydroxyapatite-Coated Collar in Megaprostheses

**DOI:** 10.3390/jfb17060291

**Published:** 2026-06-09

**Authors:** Hariharan Triplicane Dwarakanathan, Natalie Green, Thomas Haider, Iosif Pagkalos, Guy Morris, Rajesh Botchu, Lee M. Jeys

**Affiliations:** 1 Department of Orthopaedic Oncology, Royal Orthopaedic Hospital NHS Foundation Trust, Birmingham B31 2AP, UK; natalie.green5@nhs.net (N.G.); thomas.haider@me.com (T.H.); iosif.pagkalos@nhs.net (I.P.); guy.morris@nhs.net (G.M.); lee.jeys@nhs.net (L.M.J.); 2 Department of Orthopaedics and Trauma Surgery, General Hospital of Vienna, Medical University of Vienna, Waehringer Guertel 18-20, 1090 Vienna, Austria; 3 Department of Radiology, Royal Orthopaedic Hospital NHS Foundation Trust, Birmingham B31 2AP, UK; rajesh.botchu@nhs.net

**Keywords:** tumour endoprosthesis, osseointegration, 3D-printed implants, hydroxyapatite coating, orthopedic reconstruction, porous titanium, implant loosening, bone ongrowth, revision arthroplasty, extracortical fixation

## Abstract

This is a retrospective, single-center cohort study comparing the long-term radiographic osseointegration and aseptic loosening between a 3D-printed EPORE^®^ collar and a prior generation HA-coated collar in endoprosthetic reconstructions. Achieving stable bone integration in endoprosthetic reconstructions remains challenging, with hydroxyapatite (HA)-coated collars being the only option available in the past. Earlier studies from our center have shown reliable and accelerated osseointegration at the bone–collar interface using a novel highly porous 3D-printed EPORE^®^ collar system compared to a previously used HA-coated collar. **Methods**: Twenty-eight patients who underwent an implantation of endoprostheses utilizing the novel 3D-printed EPORE^®^ collar system were case-matched to 24 patients who had previously undergone surgeries using a HA-coated collar. The mean age at surgery was 65.2 years (range: 17–95 years). Patients in the HA-coated collar group had a mean age of 63.8 years (range: 17–86 years), while those in the 3D-printed collar group had a mean age of 66.7 years (range: 32–95 years), with no statistically significant difference between groups (*p* = 0.876). A minimum radiological and clinical follow-up of 2 years was available in all included cases. Osseointegration was evaluated using postoperative plain radiographs in two planes based on a previously validated semi-quantitative score. **Results:** When aseptic loosening was used as the primary endpoint, no failures occurred in the 3D-printed EPORE^®^ group during the study period. The overall rate of stem loosening (including both aseptic and septic causes) was 7% (2/28) in the 3D-printed group and 16% (4/24) in the HA-coated group. All cases of loosening in the 3D-printed cohort were related to septic failure. This translates into a 2-year aseptic-loosening-free survival of 100% in the 3D-printed group. When the radiographic osseointegration was analyzed as the endpoint, the rate of successful osseointegration was significantly higher in the 3D-printed group (92.9%, 26/28; 95% CI 76.5–99.1%) compared with the HA-coated group (70.8%, 17/24; 95% CI 48.9–87.4%; *p* = 0.04). The distribution of ongrowth scores also differed significantly between groups. The highest ongrowth score (grade 4) was achieved in 82.14% of 3D-printed implants (23/28; 95% CI 63.1–93.9%), compared with 37.5% of HA-coated implants (9/24; *p* = 0.0002). The time to achieve grade 4 ongrowth was significantly shorter in the 3D-printed cohort, with a median of 470 days (IQR 360–610), compared with 1482 days (IQR 1020–1860) in the HA-coated group (*p* < 0.0001). In addition, patients in the 3D-printed implant group had a significantly higher mean body mass index compared with the HA-coated group (32.51 vs. 28.36, *p* = 0.01). **Conclusions**: These results show that the novel highly porous 3D-printed EPORE^®^ collars reduce aseptic loosening and accelerate extracortical bridging in endoprosthetic replacements. This benefit persisted even in higher BMI or revision contexts when compared to the previously used HA-coated collars.

## 1. Introduction

Achieving consistent and durable osseointegration at the bone–prosthesis interface remains a major challenge in endoprosthetic reconstruction [1,2]. Successful osseointegration is essential to reduce aseptic loosening rates by ensuring the effective transmission of mechanical stresses across the bone–implant interface [3,4]. While hydroxyapatite (HA)-coated collars have traditionally been used to promote integration, clinical outcomes have been variable, with relatively low and unpredictable osseointegration rates [5,6,7].

**Figure 1:**
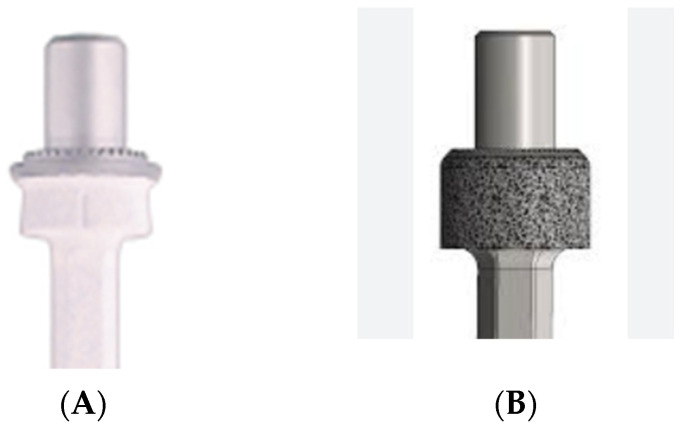
Surface morphology of (**A**) HA-coated and (**B**) 3D-printed EPORE collars.

The hydroxyapatite (HA) coating mimics natural bone minerals, promoting direct bone bonding by releasing calcium and phosphate ions that form a biological apatite layer on the implant surface. This bioactive surface enhances protein adsorption and osteoblast adhesion, leading to faster osseointegration without an intervening fibrous tissue layer. Recent advancements in additive manufacturing have opened new possibilities for implant design [8,9]. In particular, 3D printing technologies now allow for the fabrication of implants with highly controlled porous architectures that closely replicate the morphology of cancellous bone [10]. One such innovation is the EPORE^®^ collar system, which incorporates a titanium scaffold with 100–500 μm interconnected pores, a calcium–phosphate coating and a trabecular-like structure formed by 350 μm titanium rods. These collars are demonstrated later in Figure 1.

This design is supported by a growing body of evidence suggesting that such microarchitectures enhance biological fixation [11]. Studies of similar porous implants in revision arthroplasty have reported osseointegration rates of 78–89% at 2-year follow-ups [12,13]. Biomechanical evaluations suggest some advantages of this design rationale, including a greater initial stability through mechanical interlocks, enhanced osteoconduction due to optimized pore geometry and long-term fixation through progressive bone ingrowth [14].

Our initial findings at the one year follow-up confirmed these theoretical benefits, showing faster and more consistent osseointegration with porous collars compared to conventional HA-coated designs [15]. The primary outcomes of this study were an assessment of aseptic loosening and evidence of radiographic osteointegration, defined as grade 3 and above. What is novel about this study is the evaluation of these outcomes with a longer follow-up, in a higher-risk case mix, and with a systematic analysis of the time to osseointegration, compared with HA-coated collars, thus providing a comprehensive assessment of both the radiographic and clinically relevant implant performance.

## 2. Methods

This retrospective study included 52 patients (28 in the 3D EPORE group and 24 in the HA collar group) with a minimum follow-up of 2 years. All patients had a regular follow-up with X-rays performed in at least 2 planes post-op. In all cases, fellowship-trained orthopedic surgeons with longstanding experience in the use of endoprostheses for oncology and revision arthroplasty performed the surgery at a single specialist centre. This retrospective analysis was approved by the Royal Orthopedic Hospital and follows up patients from a previous study. All data were anonymized prior to analysis, and no patient-identifiable information was included. Patients provided consent for their data to be used in this study as part of the standard consent process. A waiver had already been obtained for the initial study. Given the retrospective design and relatively small sample size, no formal a priori power calculation was performed. This study should therefore be interpreted as exploratory, with findings requiring validation in larger cohorts.

Osseointegration was graded using a previously published semi-quantitative scale [5]. Bone ongrowth was assessed by analyzing two bone–collar interfaces on the ap (anteroposterior) and lateral X-rays, respectively. This scale grades the four bone–collar interfaces (two per view) as follows: grade 1 (no visible ongrowth on any interface), grade 2 (bony overgrowth with persistent gap), grade 3 (osseointegration on one or two interfaces), and grade 4 (visible ongrowth on at least three interfaces). The radiographic assessment of osseointegration was performed by two independent, fellowship-trained surgeons who were blinded to implant type, fixation method, and clinical outcomes. Inter-observer reliability for the semi-quantitative ongrowth scale was excellent, with an intraclass correlation coefficient (ICC) of 0.913 {95% confidence interval (CI): 0.847 to 0.952} and a weighted Cohen’s kappa of 0.857 (95% CI: 0.758 to 0.956), indicating almost perfect agreement. The exact agreement between raters was 88.5% (46 of 52 cases). Any discrepancies in scoring were resolved by a consensus review to determine the final grade for analysis.

## 3. Statistical Analysis

Continuous variables were tested for normality with the Shapiro–Wilk test. Normally distributed variables are reported as mean ± standard deviations and were compared using the independent samples “*t*-test”; non-normally distributed variables are reported as the median (IQR) and compared using the Mann–Whitney U test. Categorical variables were compared using Chi-squared or Fisher’s exact tests. A two-sided alpha of 0.05 defined significance.

A time-to-event analysis using the Kaplan–Meier method was employed for the implant survival free of aseptic loosening and time to grade 4 radiographic ongrowth, with group comparisons made using the log-rank test. Survival estimates are reported with 95% confidence intervals (CIs). The precision of the 0% aseptic loosening rate in the 3D-printed group is reported with its exact 95% upper confidence bound. The inter-observer reliability for radiographic grading was assessed with the intraclass correlation coefficient (ICC) and weighted Cohen’s kappa (κ). A *p* value < 0.05 was considered statistically significant for all employed tests. The data was analyzed and visualized using R software, (version 4.5.0; R Foundation for Statistical Computing, Vienna, Austria). SPSS (Version 25.0, IBM, Armonk, NY, USA) and GraphPad Prism (Version 9, GraphPad Software, La Jolla, CA, USA).

## 4. Results

We included 52 patients in this study, with 24 patients (46.15%) receiving HA-coated prostheses and 28 patients (53.84%) receiving 3D-printed prostheses. Preoperative and perioperative demographic data are presented in Table 1. The cohort comprised 24 females (46.15%) and 28 males (53.84%), with no statistically significant difference in the sex distribution between groups (HA-coated: 58.33% female; 3D-printed: 35.71% female; *p* = 0.11). The median age of the overall cohort was 69 years {interquartile range, (IQR), 57–78}. The median age was comparable between the HA-coated collar group {69 years, (IQR) 56–77} and the 3D-printed collar group (70 years, IQR 57–78), with no statistically significant difference between groups (*p* = 0.51). However, the patients in the 3D-printed collar group had a significantly higher BMI compared with the HA-coated group (median 32.5 kg/m^2^, range: 20.1–38.7 vs. 28.4 kg/m^2^, range: 17.6–37.0; *p* = 0.01). The demographic characteristics were otherwise balanced, with no further statistically significant differences observed between cohorts (Table 1).

The Charlson Comorbidity Index (CCI) served as a valuable tool to objectively quantify and compare baseline comorbidity burdens between our study groups [16]. As a validated and widely adopted metric in orthopedic and oncologic research, the CCI provided a standardized approach to assess prognostic comorbidities that could influence surgical outcomes. Our analysis revealed well-balanced cohorts, with comparable median CCI scores between the HA-coated(3.6, range 1–6) and 3D-printed collar groups (3.96, range 0–8, *p* = 0.43). This similarity in comorbidity profiles strengthens the validity of our comparative outcome analysis, as it suggests that any differences in osseointegration are unlikely to be driven by disparities in patients’ underlying health status. There was a significant difference in the anatomical location of implants (*p* = 0.016). The HA-coated collar was used more commonly in proximal femur replacements (66.7%), while the 3D-printed group had more distal femur implants (64.3%). Proximal tibia replacements were rare overall.

The indications for surgery were broadly comparable between the two cohorts, with no statistically significant difference observed (*p* = 0.21). However, there was a trend toward a higher proportion of revision arthroplasty cases in the 3D-printed collar group (42.86% vs. 25%) and a lower proportion of primary malignancy cases (10.71% vs. 29.16%) compared with the HA-coated cohort. Periprosthetic joint infections remained a major indication for surgery in both groups (35.71% vs. 41.66%).

**Figure 2:**
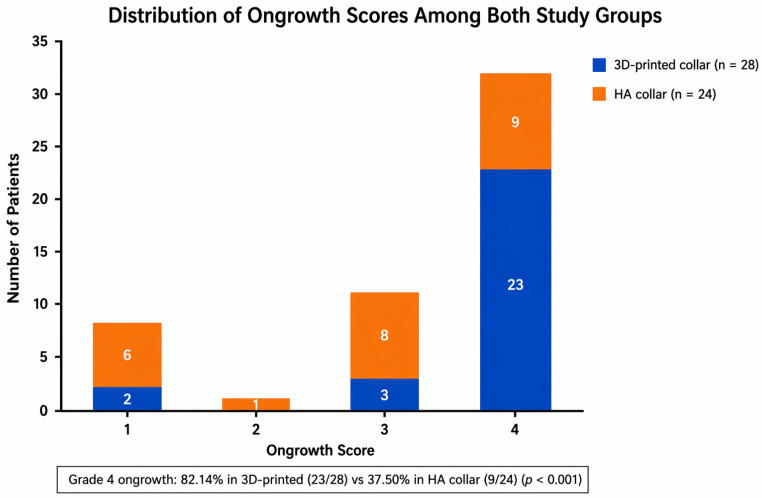
The distribution of ongrowth scores between both study groups. The distribution of ongrowth scores differed significantly between the two groups. The HA collar cohort had a higher proportion of grade 1 and grade 3 cases, whereas the 3D-printed collar group showed a greater prevalence of grade 4 osteointegration. The difference in the percentage of patients achieving grade 4 ongrowth was statistically significant {82.14% in 3D-printed versus 37.5% in HA collar (*p* < 0.001)}, indicating superior bone integration in the 3D-printed collar group compared to the HA collar group.

These patterns are clinically relevant, as revision arthroplasty and infection-related reconstructions are typically associated with compromised bone stock, impaired biology, and a higher risk of failure. The fact that the 3D-printed collar cohort included a greater proportion of such challenging cases, yet still demonstrated superior osseointegration and no aseptic loosening, strengthens the argument that the observed benefits are attributable to the implant design rather than the case selection alone. The distribution of ongrowth scores differed significantly between the two groups. The HA-collar cohort had a higher proportion of grade 1 and grade 3 cases, whereas the 3D-printed collar group showed a greater prevalence of grade 4 osseointegration. The difference in the percentage of patients achieving grade 4 ongrowth was statistically significant (82.14% in the 3D-printed group versus 37.5% in the HA-collar group; *p* < 0.001), indicating superior bone integration in the 3D-printed collar group compared to the HA-collar group. Post-operative outcomes (Table 2 and Figure 2) were consistent with these findings, with the 3D-printed group demonstrating faster time-to-integration and no cases of aseptic loosening during follow-up.

**Figure 3:**
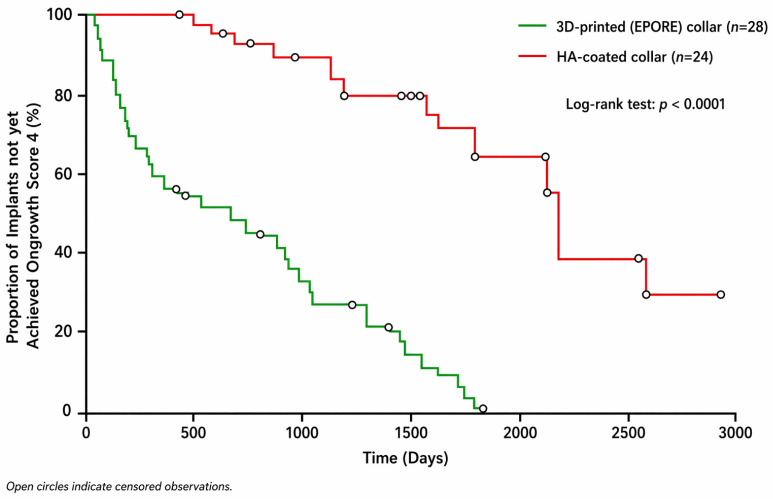
A KM curve showing the time to ongrowth score 4 distributions between two groups. The Kaplan–Meier analysis demonstrated a significant difference in the time to achieve ongrowth scores of 4 between the two groups, with the 3D-printed EPORE collar group showing faster and more frequent ongrowth attainment compared to the HA collar group (log-rank *p* < 0.05). This suggests enhanced osseointegration or earlier bone formation in the 3D-printed EPORE collar cohort.

**Figure 4:**
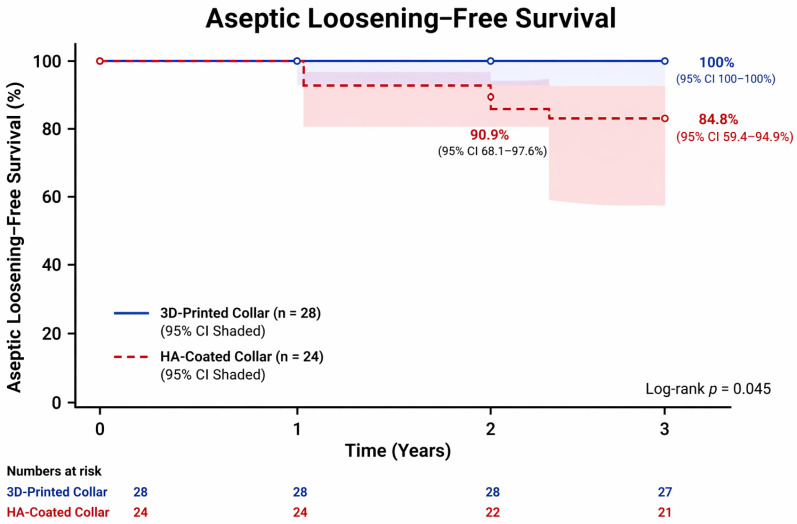
Kaplan–Meier estimates of aseptic-loosening-free survival at 1-, 2-, and 3-year intervals for HA-coated and 3D-printed collar groups. Values are presented as percentages with 95% confidence intervals (CIs). Survival analysis was performed using the Kaplan–Meier method. The 3D-printed collar group demonstrated 100% aseptic-loosening-free survival throughout follow-up, with no recorded aseptic failures.

Kaplan–Meier analysis revealed a significantly faster time to achieve grade-4 ongrowth in the 3D-printed EPORE collar group compared to the HA collar group (log-rank *p* < 0.05), indicating enhanced osseointegration in the 3D-printed cohort (Figure 3). Aseptic-loosening-free survival was 100% in the 3D-printed collar group throughout follow-up, with no recorded aseptic failures, compared to 37.5% achieving grade-4 ongrowth in the HA collar group (Figure 4; Table 3).

## 5. Discussion

To our knowledge, this is the first long-term follow-up study to assess the clinical results and osseointegration capacity of this novel highly porous 3D-printed collar (EPORE^®^), demonstrating superior osseointegration rates and mechanical stability with the novel design. These implants are often deployed in complex clinical settings, including oncologic resections and revision arthroplasty, where the risk of complications such as aseptic loosening remains high. Despite the inherent complexity of the cases, the 3D-printed collar achieved 92.86% osseointegration, significantly outperforming the HA-coated group (70.83%, *p* < 0.04). This aligns with our earlier findings at 1 year and reinforces the collar’s capacity to foster robust extracortical bridging, even in biologically challenging scenarios. Notably, the 3D-printed cohort exhibited faster and more complete extracortical osseointegration.

The superior performance in higher-BMI patients (mean 32.51 in 3D-printed group) challenges the conventional wisdom about obesity and implant fixation. Though there have been no formal studies, higher loosening rates have been noted in obese patients with standard arthroplasty implants [17]. Hence our results suggest that an optimized surface topography may mitigate this risk. This finding warrants further investigation given the increasing prevalence of obesity in patients.

A key limitation of this study is the non-randomized, historical control design, which resulted in a significant difference in the anatomical location of implants between groups. The HA-coated cohort consisted primarily of proximal femoral replacements, a location with a more favorable mechanical environment and historically lower loosening rates. In contrast, the 3D-printed cohort was predominantly composed of distal femoral reconstructions, which are subject to higher bending moments and lever arms, increasing the risk of mechanical failure. The finding that the 3D-printed implants demonstrated superior osseointegration and a significantly lower rate of aseptic loosening despite this unfavorable anatomical distribution is therefore particularly compelling. It suggests that the observed benefits are robust and may be attributable to the implant technology itself—specifically its enhanced porosity and potential for deeper bone ingrowth.

We found six cases of stem loosening in total, two of which were in the 3D collar group in the context of a periprosthetic joint infection requiring further surgical interventions due to the infection. The comparison of the four cases of aseptic loosening in the HA collar group suggests that effective integration reduces aseptic loosening. The absence of aseptic failures in our 3D-printed cohort (with both loosening cases being infection-related) supports the biomechanical advantage offered by the collar design, enhancing extracortical osseointegration and thereby reducing stress concentrations at the stem–cement–bone interface [18,19].

The 3D-printed collar group performed well in revision cases and revision arthroplasty as well. Three key findings warrant emphasis: First, the 3D collar’s time-to-integration advantage persisted in the long term, with 82% maintaining grade 4 osseointegration at 2 years versus 37.5% for HA-coated collars (*p* < 0.001). This aligns with biomechanical studies showing porous titanium’s superior osteoconductivity, where pore sizes of 100–500 μm promote vascular invasion and bone deposition [20,21].

Notably, our results assume significance, as the historical data suggests ≤50% osseointegration with HA collars in revision arthroplasty cases [5]. The 3D collar achieved 92% integration even in revision arthroplasty—a population where prior studies found only a 27% success rate [5]. This has important implications for managing these cases and cost savings from avoiding repeat surgeries.

The follow-up discrepancy, which was significant (925 vs. 1326 days, 3D versus HA), reflects the newer technology’s introduction timeline. However, the collars performed significantly better even within the short time frame, and the aseptic-loosening-free survival curve proves that this advantage persists with time. The anatomical distribution difference (more distal femoral replacements in the 3D-printed group) introduces potential confounding. However, as distal femur reconstructions typically experience higher mechanical stresses, the 3D-printed collar’s performance in these theoretically higher-risk cases strengthens the case for using these collars. A key difference between groups was the fixation method. All implants in the 3D-printed collar cohort were cemented, whereas the HA-coated group included both cemented (66.7%) and uncemented stems. This reflects differences based on surgeon choice rather than an intentional selection bias. However, this discrepancy introduces a potential confounding factor, as cemented fixation may influence initial implant stability and load transfer characteristics.

Despite this, the superior osseointegration observed in the 3D-printed collar group is unlikely to be explained solely by the fixation method. Extracortical osseointegration at the collar–bone interface is biologically driven and distinct from stem fixation. Furthermore, HA-coated collars have historically been used with both cemented and uncemented stems yet still demonstrate variable and often inferior rates of extracortical integration in prior studies. Therefore, while the fixation method may contribute to the overall construct stability, the observed differences in the ongrowth and time-to-integration are more plausibly attributable to the enhanced porous architecture of the 3D-printed collar.

As the use of 3D-printed collars becomes more widespread, future prospective studies with larger sample sizes are needed to validate these findings and to further evaluate functional outcomes, implant survivorship, and the independent effect of the implant design through multivariable analyses.

## 6. Conclusions

The use of a 3D-printed porous collar in endoprosthetic reconstruction demonstrates encouraging mid-term outcomes in our series. The improved implant stability enhanced the biological fixation, and a lower incidence of aseptic loosening was observed, particularly in complex revision settings. These findings suggest that this design may contribute to more durable reconstructions and better functional outcomes. Further long-term studies are warranted to confirm its sustained benefits and broader applicability.

## Figures and Tables

**Table 1:** Demographic characteristics of both study cohorts.

Variables	Overall (*n* = 52)	HA-Coated (*n* = 24)	3D-Printed (*n* = 28)	*p* Value
Female (*n*%)	24 (46.15)	14 (58.33)	10 (35.71)	0.16
Male (*n*%)	28 (53.84)	10 (41.67)	18 (64.28)	
Age, median (IQR)	69 (57–78)	69 (56–77)	70 (57–95)	0.51
BMI, median (IQR)	30 (17.6–38.7)	28.36 (17.6–37)	32.51 (20.1–38.7)	0.01
CCI, mean (range)	3.8 (0–8)	3.6 (1–6)	3.96 (0–8)	0.43
Previous revision surgery (*n*%)	38 (73.1)	16 (66.67)	22 (78.57)	0.36
Peri-chemo (*n*%)	7 (13.46)	3 (12.5)	4 (14.29)	1.00
RT (*n*%)	1 (0.19)	1 (0.41)	0 (0)	0.46
Indication (*n*%)				0.21
Revision arthroplasty	18 (34.62)	6 (25)	12 (42.86)	
Periprosthetic infection	20 (38.46)	10 (41.66)	10 (35.71)	
Primary malignancy	10 (19.23)	7 (29.16)	3 (10.71)	
Bone metastasis	4 (7.69)	1 (4.16)	3 (10.71)	
Type of implant (*n*%)				0.016
Proximal femur	25 (48.07)	16 (66.66)	9 (32.14)	
Distal femur	26 (50)	8 (33.33)	18 (64.28)	
Proximal tibia	1 (1.9)	0	1 (0.3)	
Cement fixation (*n*%)	44 (84.61)	16 (66.66)	28 (100)	0.002

**Table 2:** Postoperative outcomes.

Variables	Overall (*n* = 52)	HA-Coated (*n* = 24)	3D-Printed (*n* = 28)	*p* Value
Follow-up days, mean (SD)	1125 (=/−668)	1326 (±525)	925 (±2 87)	0.002
Osseointegration yes/no (*n*%)	43 (82.69)	17 (70.83)/7 (29.16)	26 (92.86)/2 (0.71)	0.04
Ongrowth score (*n*%)				
1	8 (15.3)	6 (25)	2 (7.14)	0.12
2	1 (1.9)	1 (4.1)	0 (0)	0.46
3	11 (21.15)	8 (33.3)	3 (10.7)	0.08
4	32 (61.5)	9 (37.5)	23 (82.14)	0.001
Stem loosening (*n*%)	6 (11.53)	4 (16.66)	2 (7.14)	0.38

**Table 3:** Kaplan–Meir analysis of aseptic-loosening-free survival.

Time Point	HA-Coated Collar	95% CI	3D-Printed Collar	95% CI
1 year (365 days)	100%	100–100%	100%	100–100%
2 years (730 days)	90.9%	68.1–97.6%	100%	100–100%
3 years (1095 days)	84.8%	59.4–94.9%	100%	100–100%

## Data Availability

Due to ethical and privacy restrictions imposed by the Royal Orthopaedic Hospital and in compliance with UK data protection laws (including the UK GDPR and NHS confidentiality guidelines), the raw data supporting the findings of this study cannot be made publicly available. The data could contain potentially identifiable patient information from a retrospective review of clinical records.

## References

[1] Jeys L.M., Kulkarni A., Grimer R.J., Carter S.R., Tillman R.M., Abudu A. (2008). Endoprosthetic reconstruction for the treatment of musculoskeletal tumors of the appendicular skeleton and pelvis. J. Bone Jt. Surg. Am..

[2] Myers G.J.C., Abudu A.T., Carter S.R., Tillman R.M., Grimer R.J. (2007). Endoprosthetic replacement of the distal femur for bone tumours: Long-term results. J. Bone Jt. Surg. Br..

[3] Torbert J.T., Fox E.J., Hosalkar H.S., Ogilvie C.M., Lackman R.D. (2005). Endoprosthetic reconstructions: Results of long-term followup of 139 patients. Clin. Orthop. Relat. Res..

[4] Coathup M.J., Sanghrajka A., Aston W.J., Gikas P.D., Pollock R.C., Cannon S.R., Skinner J.A., Briggs T.W.R., Blunn G.W. (2015). Hydroxyapatite-coated collars reduce radiolucent line progression in cemented distal femoral bone tumor implants. Clin. Orthop. Relat. Res..

[5] Davies B., Kaila R., Andritsos L., Stephens C.G., Blunn G.W., Gerrand C., Gikas P., Johnston A. (2021). Osteointegration of hydroxyapatite-coated collars in cemented massive endoprostheses following revision surgery. Bone Jt. Open.

[6] Sankar B., Refaie R., Murray S.A., Gerand C.H. (2012). Bone growth on hydroxyapatite collars in massive tumour endoprostheses. Orthop. Proc..

[7] Tanzer M., Turcotte R., Harvey E., Bobyn J.D. (2003). Extracortical bone bridging in tumor endoprostheses. Radiographic and histologic analysis. J. Bone Jt. Surg. Am..

[8] England T., Pagkalos J., Jeys L., Botchu R., Carey Smith R. (2021). Additive manufacturing of porous titanium metaphyseal components: Early osseointegration and implant stability in revision knee arthroplasty. J. Clin. Orthop. Trauma.

[9] Denehy K.M., Abhari S., Krebs V.E., Higuera-Rueda C.A., Samuel L.T., Sultan A.A., Mont M.A., Malkani A.L. (2019). Metaphyseal Fixation Using Highly Porous Cones in Revision Total Knee Arthroplasty: Minimum Two Year Follow up Study. J. Arthroplast..

[10] Meng M., Wang J., Huang H., Liu X., Zhang J., Li Z. (2023). 3D printing metal implants in orthopedic surgery: Methods, applications and future prospects. J. Orthop. Transl..

[11] Mumith A., Coathup M., Chimutengwende-Gordon M., Aston W., Briggs T., Blunn G. (2017). Augmenting the osseointegration of endoprostheses using laser-sintered porous collars: An in vivo study. Bone Jt. J..

[12] Shichman I., Oakley C., Willems J.H., van Hellemondt G.G., Heesterbeek P., Rozell J., Marwin S., Schwarzkopf R. (2023). Novel metaphyseal porous titanium cones allow favorable outcomes in revision total knee arthroplasty. Arch. Orthop. Trauma Surg..

[13] Brown N.M., Bell J.A., Jung E.K., Sporer S.M., Paprosky W.G., Levine B.R. (2015). The Use of Trabecular Metal Cones in Complex Primary and Revision Total Knee Arthroplasty. J. Arthroplast..

[14] Wang X., Zhang D., Peng H., Yang J., Li Y., Xu J. (2023). Optimize the pore size-pore distribution-pore geometry-porosity of 3D-printed porous tantalum to obtain optimal critical bone defect repair capability. Biomater. Adv..

[15] Haider T., Pagkalos I., Morris G., Parry M.C., Jeys L.M. (2023). Early radiographic osseointegration of a novel highly porous 3D-printed titanium collar for megaprostheses compared to a previous generation smooth HA-coated collar. Arch. Orthop. Trauma Surg..

[16] Charlson M.E., Pompei P., Ales K.L., MacKenzie C.R. (1987). A new method of classifying prognostic comorbidity in longitudinal studies: Development and validation. J. Chronic Dis..

[17] Deo S.D., Jonas S.C., Frcs J.J. (2022). Early Aseptic Tibial Loosening in Total Knee Replacement—A Gender and Obesity Related Complication. Ann. Orthop. Rheumatol..

[18] Ward W.G., Johnston K.S., Dorey F.J., Eckardt J.J. (1993). Extramedullary porous coating to prevent diaphyseal osteolysis and radiolucent lines around proximal tibial replacements. A preliminary report. J. Bone Jt. Surg. Am..

[19] Coathup M.J., Batta V., Pollock R.C., Aston W.J., Cannon S.R., Skinner J.A., Briggs T.W.R., Unwin P., Blunn G.W. (2013). Long-term survival of cemented distal femoral endoprostheses with a hydroxyapatite-coated collar: A histological study and a radiographic follow-up. J. Bone Jt. Surg. Am..

[20] Jung A., Jang J., Ban H.Y., Kim H.J., Gweon B., Lim D. (2025). Enhanced biomechanical and biological performance of titanium scaffolds with gradient in pore sizes. J. Mater. Res. Technol..

[21] Stich T., Alagboso F., Křenek T., Kovářík T., Alt V., Docheva D. (2022). Implant-bone-interface: Reviewing the impact of titanium surface modifications on osteogenic processes in vitro and in vivo. Bioeng. Transl. Med..

